# Francis Darwin (1848–1925): the biologist in the shadow of father Charles

**DOI:** 10.1080/15592324.2025.2509264

**Published:** 2025-05-30

**Authors:** Ulrich Kutschera, František Baluška

**Affiliations:** AK Evolutionsbiologie, 79104 Freiburg i. Br. Germany & I-Cultiver, Inc, San Francisco, CA, USAkutscherau@gmail.com; Institut für Zelluläre und Molekulare Botanik (IZMB), Universität Bonn, Germany

**Keywords:** Darwin, Sachs, plant movements, root-brain hypothesis, stomata, water

## Abstract

This year marks the 100th anniversary of the death of Sir Francis Darwin (1848–1925), the biologist/naturalist, musicologist, and biographer/philosopher of science in the shadow of father Charles (1809–1882). Francis was the seventh child and third son of the British couple. Here, we present a concise biography of Francis Darwin, based on his neglected “Recollections” published in his book *Springtime and other Essays* (1920). We summarize the scientific legacy of the younger Darwin with a focus on the “root-brain-hypotheses”, as described in the 1880- “Darwin & Darwin book *The Power of Movements in Plants*, his extensive work on water-loss (transpiration) in plants via the stomata, and the Francis D.-papers on music instruments. In addition, we shed new light on the well-known “Darwin-Julius Sachs”-controversy of the 1880s. Finally, we document that the Biologist Francis Darwin was also a musician and polymath, with broad expertise in the natural sciences and the humanities alike.

## Introduction

Ten years ago, we published a short article in Plant Signaling & Behavior entitled “Julius Sachs (1832–1897) and the Unity of Life”. In this essay, the contents of the *Handbuch der Experimental-Physiologie der Pflanzen* (Experimental Physiology of Plants), published in 1865 by the then 33-year-old German “Father of Plant-Physiology”, were summarized.

However, one topic of minor importance was not explicitly addressed: the well-known and widely prevalent endogenous movements observed in most species of land plants (embryophytes). These processes are very similar to those documented in the stem of climbing plants, such as *Bryonia dioica*: The axis bends in successive steps in all directions, with the result that the shoot tip revolves.

In the *Handbuch* of 1865, Sachs^1^ coined the term “revolving movements” to describe this ubiquitous phenomenon.

Fifteen years later, Charles Darwin (1809–1882), in his book *The Power of Movements in Plants*, replaced this “Sachsian” terminology by coining the phrase “Circumnutation” to denote these processes.^2^

According to the resulting “Darwinian circumnutation theory”, all organ movements in the vegetable kingdom, i.e., tropisms, nastic processes etc., originated, with modifications, from this evolutionarily ancient “archetype” of plant movements.

**Figure 1.**
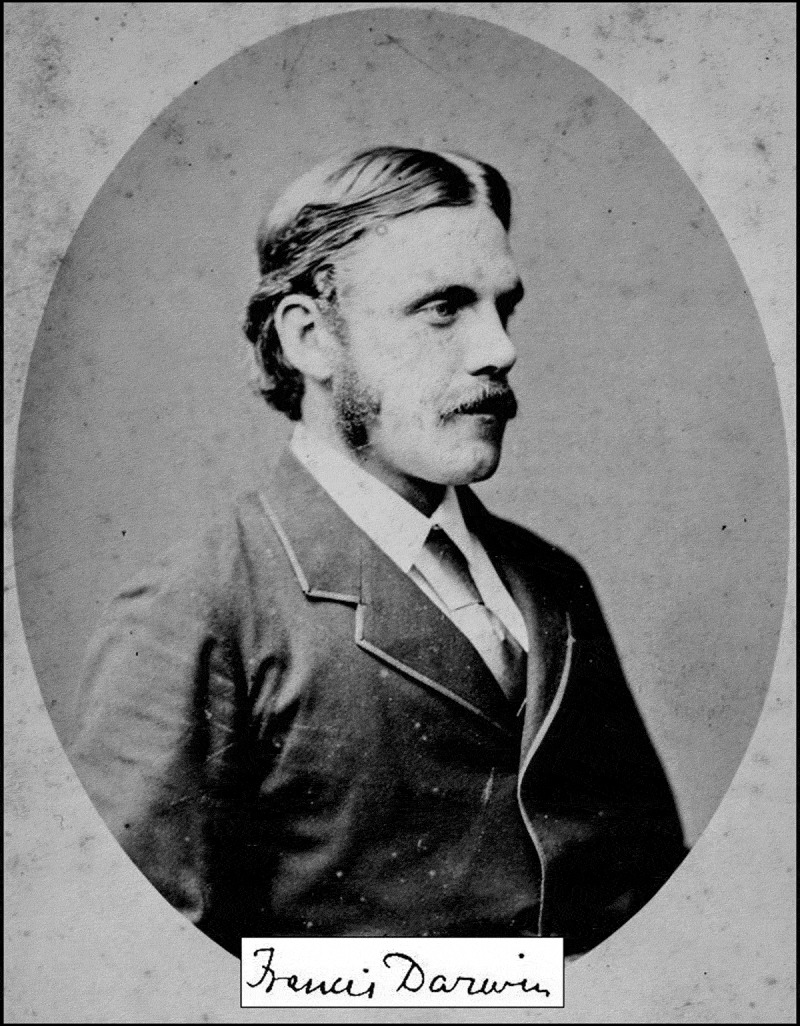
Portrait of Sir Francis Darwin (1848–1925), the only child of Charles and Emma who had inherited the drive to become a biologist from his father, and a strong interest in classical music from his mother, who was a gifted pianist (adapted from ref. 14).

The extensive experimental work on which *The Power of Movements* rests was not carried out by “father Charles” alone: Only with the help of his son Francis Darwin (1848–1925) (Figure 1) was it possible to collect such a fundus of original observations as documented in this 582-pages “father-and son-book” published in 1880 (ref. 2).

In this *Editorial*, commemorating the 100th anniversary of Francis Darwin’s death, we describe the life, achievements, and lasting legacy of this eminent scientist. Our goal is to show that the “biologist in the shadow of father Charles” was not only a neglected botanist, but rather a gifted polymath whose expertise comprised the biological sciences, history-philosophy, and musicology alike.

## Francis Darwin: short biography – based on his recollections

Francis was the seventh child and third son of father Charles and his wife Emma Darwin (1808–1896), who was a first cousin of her husband. It should be noted that Father Charles was aware of the possible negative genetic consequences of this marriage among close relatives with respect to the health of their children—a topic we will not discuss in this contribution.

**Figure 2.**
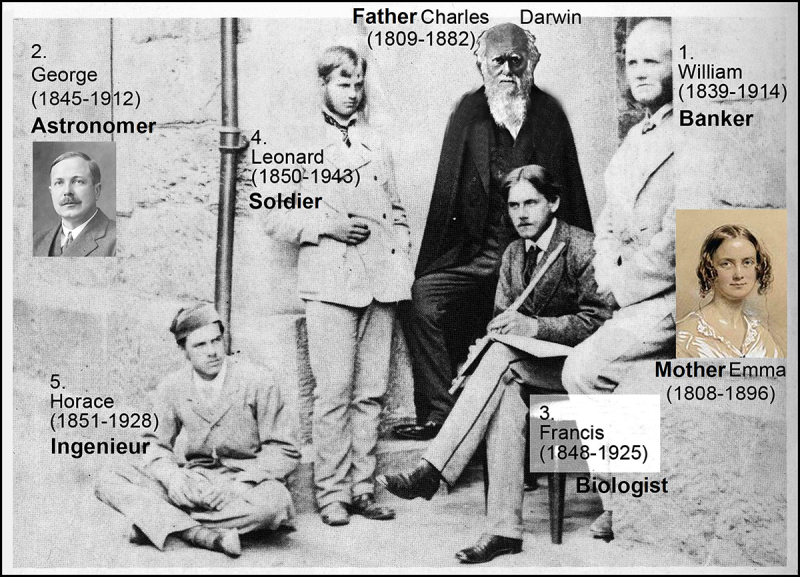
Father Charles and mother Emma Darwin with their five sons: William, George, Francis (with flute), Leonard and Horace. Only Francis became a biologist (like his father) and a musician (like his mother) (adapted from historical images).

In his book *Springtime and other Essays*, Francis Darwin (1920)^3^ described his childhood as follows (Figure 2):

“I was born at Down on 16th August 1848: I was christened at Malvern … , and wish that I had received some other name. I was never called Francis, and I disliked the usual abbreviation Frank, while Franky or Frankie seemed to me intolerable. I also considered it a hardship to have but one Christian name. Our parents began by giving two names to the elder children …

### Aversion to religion and the kind father

Concerning his attitude toward Christianity, F. Darwin outlined this topic in the following words: “I have no idea at what age we began to go to church, but I have a general impression of unwillingly attending divine service for many boyish years … We certainly were not brought up in Low Church or anti-Papistical views, and it remains a mystery why we continued to do anything so unnecessary and uncomfortable.

I have a general impression of coming out of church cold and hungry … My recollection is that we often went only to the afternoon service, which we preferred for its brevity. I have a clear recollection of our delight when, on rainy Sundays, we escaped church altogether. A feature that distinguished Sunday from the rest of the week was our singular custom of having family prayers on that day only. … On Sundays we wore our best jackets, but I think that, when church was over, we put on our usual tunics or blouses of surprising home-made fit. But I clearly remember climbing (in my Sunday clothes) a holly-tree on a damp Christmas Day, and meeting my father as I descended green from head to foot. I remember the occurrence because my father was justly annoyed, and this impressed the fact on me, since anything approaching anger was with him almost unknown.”

### Servants, butlers and Gardeners

Then, F. Darwin (1920)^3^ described his life as a juvenile at Down House: “In the early days of which I was speaking, we had schoolroom tea with our governess, while our parents dined in peace at about 6.30.” … We were fortunate in having a set of simple, kindly, old-fashioned servants with whom we could be on friendly terms. Thus, it happens that recollections cluster about the kitchen and pantry. I have a vague remembrance of a Welsh cook, Mrs. Davis, who was very kind to us … We certainly could generally extract gingerbread and other good things from Daydy, as we called Mrs. Davis. The butler, Parslow, was a kind friend to us all our lives. I do not remember being checked by him except in being turned out of the dining-room when he wanted to lay the table for luncheon, or being stopped in some game which threatened the polish of the sideboard, of which he spoke as though it were his private property. … It was good to see him on Christmas Day: with how great an air would he enter the breakfast-room and address us: ‘Ladies and Gentlemen, I wish you a happy Christmas, etc. etc.’ I am afraid he got but a sheepish response from us. Among the outdoor servants there were three whom I remember well. There was Brooks, the general outdoor man, who acted as gardener, cowman, etc. He had dark eyes and a melancholy, morose face. … Brooks lived in a cottage close to the cow-yard, with his wife … The under-gardener, Lettington … , was a kindly person and a great friend of mine. It was he who taught me to make whistles in the spring and helped me with my tame rabbits. He also showed me how to make brick-traps for small birds, and a more elaborate trap made of hazel twigs. …

To return to Lettington. It was he who helped my father in his experiments on the crossing of plants: he lived to a great age, dying as a pensioner many years later. My father used to tell with amusement how Lettington never failed to remind him of a bad prophecy: ‘Yes, sir, but you said so-and-so would happen.’ The third outdoor man was Thomas Price, generally known as the Dormouse on account of his somnolent manner of working …. He was a bachelor and spent more than was wise on beer. … ”

### George, Francis, Leonard

Next, F. Darwin^3^ recounts his relationships with his brothers:

“To return to my childhood: I came between George and Leonard, and was a companion to both of them, but I do not think we made a trio as Leonard and Horace and I did more or less (Figure 2). I seem to remember a great deal of purposeless wandering with my younger brothers; but with George, playing was an organized affair in which I was an obedient subordinate … Our chief game was playing at soldiers; we had toy guns to which home-made wooden bayonets were fixed, knapsacks, and I think shakos; whether we had any uniform coats I cannot remember. …

Another pursuit was walking on stilts, of which we had two kinds; on the smaller ones even girls had been known to walk, but of the larger (which I remember as of imposing height) only the male sex was capable. The garden at Down was originally a bare and windy wilderness, but our parents constructed mounds of raw red clay …

### Life-exclusively within the family

Son Francis complained about the fact that he and his brothers were raised isolated from other children: “It is curious to remember how solitary our life was. We had literally no boy-friends in the whole neighborhood; there were plenty of boys within reach but we never amalgamated with them, and were, I imagine, despised by them as outside the pale of Eton-dom. No opportunity was made for us to learn to shoot; I used to wander with a gun and shoot an occasional hare and various blackbirds, but I never had even the meanest skill, and after suffering miseries of shame at one or two shooting-parties I am glad to think I gave it up. Fishing there was none in our dry country, and it was only very much later, on the beautiful Dovey in North Wales … ”

Concerning Riding, Francis Darwin^3^ wrote that … “The best practice I had as a boy was riding twice or thrice a week (from perhaps my tenth to my twelfth year) to Mr. Reed, Rector of Hayes, to be taught Latin and a little arithmetic. Our ponies were shaggy, obstinate little beasts, who had the strongest possible dislike of their duties.”

### From school to Trinity College

In the following section, F. Darwin (1920)^3^ outlined his career as a pupil and student: “When I was twelve years old, *i.e*. in the summer of 1860, I went to the Grammar School at Clapham kept by Rev. Charles Pritchard. I was two years under Pritchard, and when he left I remained under his successor, Rev. Alfred Wrigley, until I went up to Trinity College, Cambridge-1866. Wrigley had none of the force of Pritchard, nor had he, I fancy, his predecessor’s gift of teaching. Mathematics formed a great part of our curriculum, and for these I had no turn. I am, however, grateful to Wrigley for having made me work out a great many logarithmic calculations, which had to be shown up (as he expressed it) in a ‘neat, tabular form.’ From school I went to Trinity College, Cambridge.

I hardly made any permanent friends till my second year … . I am glad to think that my undergraduate friends (except those removed by death) are still my friends.

Among the Dons who were friendly to students of natural science the first place must be given to Alfred Newton (1829–1907), the Professor of Zoology, who most kindly invited us to come to his rooms in Magdalene any and every Sunday evening. There we smoked our pipes and enjoyed ourselves thoroughly. We had the advantage of meeting older members of the University. It was in this way I became acquainted with G. R. Crotch, of St John’s, who was an assistant in the University Library. He was a strikingly handsome man with a long silky beard and wonderful eyes. His passion was Entomology, and he had a great knowledge of the Coleoptera, and used sometimes to take me out beetle-catching, but I never became a collector. … He finally gave up his librarianship and went off beetle-catching to the United States, where he died … John Willis Clark (1833–1910) (who afterward became Registrary of the University) was then Curator of the New Museums, and encouraged me to work in his department, and I well remember my pride when my preparation of a hedgehog’s inside was added to the Museum. J. W. Clark was the kindest of men, and I, like many another undergraduate, used to dine with him and his mother at Scrope House. … I had left to the last the man whose kindness toward me as an undergraduate I valued most highly, and whose friendship it is still my good fortune to possess – I mean Henry Jackson (1839–1921), now Professor of Greek, but at that time a Trinity lecturer. I have an image of him walking up and down his room in Neville’s Court with a pipe in his mouth …

### Academic studies

I must now return to my more serious employments. It was at the suggestion of Edward C. Stirling (1848–1919) that I became a medical student and began to work for the Natural Sciences Tripos. In order to get more time for the last-named examination I kept my small stock of mathematics simmering as it were, and managed (without giving much time to the subject) to get a mathematical degree as fifth among the Junior Optimes in 1870. I had the pleasure of being coached for this examination by James Stuart – the only man, I imagine, who ever made mathematics entertaining and even amusing to an unmathematical pupil.

I then had a clear year in which I could devote myself to Natural Science. I did not succeed in finding a coach who was of any use to me. But in Comparative Anatomy I did a fair amount of undirected work: in this way I dissected a good many creatures such as slugs and snails and freshwater mussels, dragonflies, etc.”

These recollections reveal that Francis Darwin, and his four brothers and two sisters, was primarily raised-educated by Servants, Butlers and Gardeners, who were employed by their parents. The role of “father Charles and mother Emma” with respect to the education of their children is unclear.

### Medical degree, histology, and assistant to father Charles

Francis described his subsequent career in the following words: “On leaving Cambridge I went to St George’s Hospital with the intention of becoming a practicising physician. But happily for me the Fates willed otherwise. The late Dr. Cavafy of St George’s Hospital urged me to learn something of Histology, and sent me to Dr. Klein, whose pupil I had the good fortune to become at the Brown Institute. … Under his guidance, I produced a paper, which served as a thesis for my M.B. degree (1875). I had another interesting experience during my time at St George’s. I used to go to the Zoological Society’s dissecting-room, where the late Dr. Garrod (the Prosector) allowed me to investigate some of the daily quota of dead animals. But it was not of any real educational value, I fancy. Still it may have helped the impetus of Klein’s teaching to suggest that medicine should be given up and that I should become the assistant to my father.”

### Back home – death of his first wife

In 1874, Francis married Amy Ruck (1850–1876). The couple had lived in a small house close to London. Unfortunately, his wife died shortly after giving birth to her son Bernard Darwin (1876–1961), who published, six years before his death, an autobiography. In this book, B. Darwin (1955) outlined, under the headline “Down and Childhood”, his early years: “My mother died when I was born and my father took me with him from his own smaller house to live with my grandfather and grandmother at Down-House” (B. Darwin 1955).^4^

Then, F. Darwin outlined his career as a Research Scientist and Lecturer: “The old nursery at Down had been turned into a laboratory, and when … I came to live in the house of my parents, they converted the billiard-room into a sitting-room for me. During the following years (1874/75) I went to work under Julius Sachs (1832–1897) at Würzburg and afterwards under Anton De Bary (1831–1888) at Strassburg. Sachs was most kind and helpful, and under his direction I contributed a small paper to his Journal *Arbeiten* (Ref. 5). I made some good friends at Würzburg – Ernst Stahl (1848–1919), who is now Professor of Botany at Jena; Kunkel, the Pharmacologist, who died young; the Finlander Frederik Elfving (1854–1942), who is now Professor of Botany at Helsingfors; and Karl v. Goebel (1855–1934), now the well-known Professor of Botany at Munich. He and I walked side by side to receive our degrees at the 1909 meeting in Cambridge. I had the great pleasure of seeing Elfving on the same occasion, and we have never ceased to correspond, though at irregular intervals. I had once the satisfaction of receiving Stahl as my guest at Cambridge. He is still Professor of Botany at Jena, and in spite of rather weak health has published a mass of good work.”

### The Julius Sachs-Darwin-controversy

**Figure 3.**
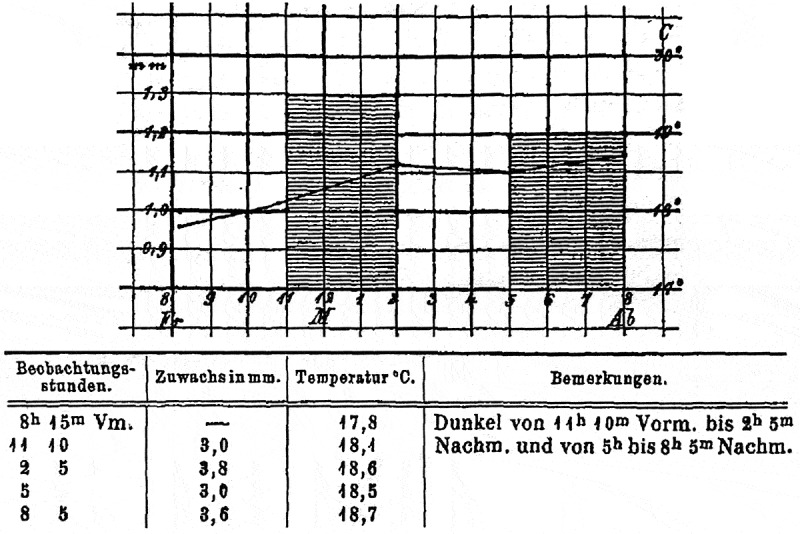
Representative experiment of Francis Darwin documenting that, in roots of mustard seedlings (*Sinapis alba*), light given from all sides inhibits the rate of organ elongation (adapted from Darwin, F.: Arbeiten Bot. Institut Würzburg 2, 521–528; 1882).

In his “Recollections” F. Darwin (1920) also recounts his negative experience with his former mentor Julius Sachs (see Kutschera and Briggs 2009^5^): “I am sorry to think that my relationship with Sachs came to an unhappy ending. I published what seemed to me a harmless paper (Figure 3), in which I criticized some of his researches. I wrote to him on the subject but received no answer. Partly on account of his silence and partly to pay a visit to a friend, I traveled to Würzburg. I found Sachs in the Botanic Garden; he seemed to wish to avoid me, but I went up to him and asked him why he was angry with me. He replied: ‘The reason is very simple; you know nothing of Botany and you dare to criticise a man like me.’ I had no opportunity of replying, for at that moment one of his co-professors addressed him, asking if he could spare a moment. ‘Very willingly, Herr Professor,’ said Sachs, and walked off without a word to me. And that was the last I saw of the great botanist. I was undoubtedly stupid, but I do not think he showed to advantage in the affair. I continued to work with my father at Down, and in spite of the advantages I gained by seeing and sharing in the work of German laboratories, I now regret that so many months were spent away from him.”

Francis Darwin’s son Bernard^4^ mentioned the “Würzburg-Time” as well as the lasting impact of the death of Amy Ruck on his father: “When I was about two … he was away working in a laboratory at Würzburg … The shock of my mother’s death had gone very deep, and I think he always had a spasm of fear when he heard of a new baby in the family to be born” …

One year after the death of father Charles (1883) Francis married his second wife, who died in 1903; the couple had one daughter. His third marriage lasted from 1913 to 1920, so that Francis Darwin survived all of his three spouses.

## The scientific legacy of Francis Darwin

Charles Darwin’s most gifted son Francis is best known as coauthor of the “Darwin & Darwin-book” *The Power of Movements in Plants*, and his edited monograph of 1885, *The Life and Letters of Charles Darwin* (vol. 1–3).^6^ As mentioned above, Francis worked as a “scientific assistant” in the laboratory of the then most famous plant physiologist/botanist of Europe, Julius Sachs at Würzburg University (Germany). During this short stint, he studied root elongation in dark-grown seedlings of white mustard (*Sinapis alba*), and discovered that all-round light depressed the rate of root growth.^7^ In the Sachs-lab Francis Darwin learned how to use sophisticated equipment and novel apparatuses to analyze plant growth and development.

In addition, he became familiar with the German language, so that he was able, in contrast to father Charles, to read the vast scientific literature published during the 19th century in the former “language of science”. Since roots of mustard seedlings bend away from light (negative phototropism) the then popular explanation that light promotes cell elongation on the irradiated side, as described by Sachs in his *Textbook of Botany*, was no longer tenable.

In 1880, F. Darwin published his results in the *Arbeiten des Botanischen Instituts Würzburg*, a journal edited by Julius Sachs.^7^ Darwin mentioned the contradiction between his findings and the “Sachsian” point of view on this topic. This may have been the major reason why Sachs was angry with Darwin.

Anyway – it is obvious that, based on the “Würzburg-experience” of son Francis (1874/75), Father Charles was now in the position to finish the “Darwin & Darwin”-book on plant movements (published in 1880, two years before the “Würzburg-Paper of Francis was printed)^2^ (Figure 3).

### Root-brain-hypothesis

The most important, and lasting part of the “Darwin & Darwin”-book was the so-called “Root-brain-hypothesis”. In their book *Power of Movements in Plants*, experiments were reported, as carried out by Francis under the guidance of his father Charles, which showed that plants perform goal-driven movements both at the shoot and root apices. While the shoot-part of their book focused on the light perception and phototropic shoot and coleoptile movements, the root part resulted in their bold statement, expressed at the very end of their book, that the root apex acts as a kind of ‘brain’ guiding movements of the adjacent root growth zones^2^ (Baluška et al. 2009, Kutschera and Niklas 2009).^8,9^ In their statement, they convey two important issues. First, the root apex is a ‘brain-like’ organ. Second, it is seated, similarly as in animals, at the anterior pole of the plant body. According to this view, plants are anchored in the soil by their ‘heads’, exposing their sexual organs for prospective pollinators at the opposite posterior pole of the plant body (Baluška et al. 2009).^8^ In 1908, in his address of the President of the British Association for the Advancement of Science, Francis Darwin went even further arguing that plants use their plant-specific sentience for the goal-directed movements of their organs (Darwin 1908a,b,c).^10–13^

### Research on graviperception and water loss

In 1932, an anonymous author (“F. F. B.”, ref. 14) published an Obituary on Francis Darwin in the *Proceedings of the Royal Society B*. Here, we summarize the pertinent work of F. Darwin with reference to this article.

In 1895, experimental work carried out in Germany revealed that perception of gravity occurs in the root tip, and five years later, the “starch-statolith-theory of gravi-sensitivity” was brought forward by Nemec and Haberlandt. Francis Darwin carried out and published original research on these topics.

In 1904, acting as president of the *Botanical Society of the British Association*, he gave an address summarizing the evidence in support of the statolith-theory that has since been corroborated many times by independent evidence, and today represents a well-established model in plant developmental physiology.^14^

Finally, between 1897 and 1916, Francis Darwin published a series of papers dealing with the control of loss of water by plants growing with their leaves in air, subjected to considerable variations of dryness and radiation (F. F. B. 1932).^15^ Like his studies on plant movements, F. Darwin’s second major research agenda was interpreted by him as an example for evolutionary adaptation.

**Figure 4.**
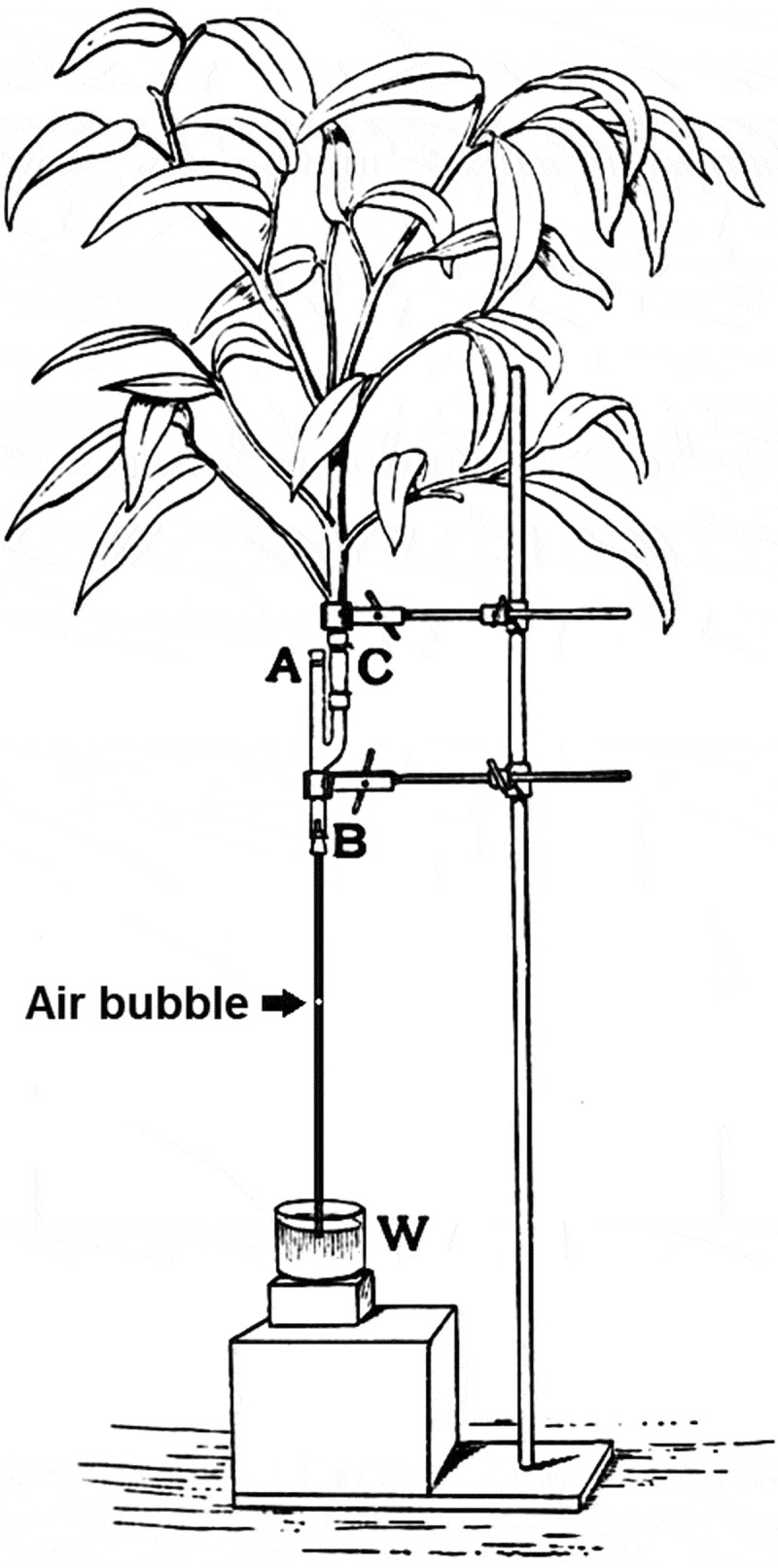
The potometer, an apparatus designed by Francis Darwin to determine water loss of a transpiring shoot, using an air-bubble in the tube to signify water movement (adapted from Darwin F. & Acton E H., Practical Physiology of Plants, 1894).

To study water-loss by the leaves, with a focus on the function of stomata, Francis Darwin invented (like Sachs in 1865)^1^ a variety of special apparatuses (Figure 4):

1. The (air-bubble) Potometer for quantification of the rate of water uptake by a transpiring shoot; 2. The (horn)-Hygroscope for estimation of the rate at which water vapor is released from the leaf surface; 3. The (electric) Leaf-Cooling Recorder for an exact determination of the rate of water evaporation from the surface of a leaf, and 4. The (diffusion path) Porometer for relative quantification of the degree of openness of the stomatal pores on the surface of a leaf.

Using these methods, Francis Darwin had “formalized” the transpiring leaf as a physical system that consists of a source (liquid water), a diffusion path (stomatal pores) and a sink (relative dry air) to study the effects of light, dryness and humidity on the rate of water-loss in land plants. Hence, F. Darwin maybe regarded as one of the neglected pioneers of “plant systems biology”. (ref. 15).

Francis Darwin was able to document that pore-opening was triggered by light, based on a nocturnal closer of the Stomata. His Porometer-data revealed that closure of the stomata reduces the loss of water at night and that re-opening begins before sunlight. In short, our modern view of the function of stomata in the regulation of water flow through the root-stem-leaf-system in typical embryophytes is based on the experimental work of the biologist in the “shadow of Father Charles” (see his last paper on this topic, published in 1916 (ref. 16) – Conclusion: ”transpiration is regulated by stomatal aperture”).

### Two books on plant biology

Finally, it should be noted that Sir Francis Darwin (knighted in 1913, an honor not received by Father Charles) wrote two important books on the physiology and biology of plants. In 1894, *The Practical Physiology of Plants* was published with a coauthor (Darwin and Acton). Since the *Handbuch der Experimental-Physiology* of Sachs (1865)^1^ had never been translated into English, “Darwin and Acton 1894” (ref. 17) became the first British book containing “recipes” as to how to manage practical classes on this topic at the College/University-level. In addition, Francis Darwin published a small book, based on lectures he had presented to medical students (*Elements of Botany*).^16^ Hence the statement of Julius Sachs (“You know nothing of Botany … ”) was wrong and unfair. However, we should keep in mind that the egocentric German Professor suffered, due to his self-imposed 16-hour-working load and isolated life-style, from mental instabilities, as documented in (ref. 14).

## History of biology and musicology

In addition to his scientific papers and contributions to the work and life of father Charles, Francis Darwin published two popular books, presenting collections of his most important shorter essays. In 1817, *Rustic Sounds*, and 3 years later, *Springtime and Other Essays* came out. Both books were published by John Murray, London.^17,16^

In *Rustic Sounds* (1817), F. Darwin presented his biographies of the scientists Francis Galton (1922–1911), Stephen Hales (1677–1761), and his brother, the astronomer George Darwin (1845–1912). Two Essays deal with education and teaching of science, and one article is dedicated to “War Music”. In *Springtime* (1920), F. Darwin summarized, in addition to his biography of Joseph Dalton Hooker (1817–1911), his Essays on Flowers and other Botanical Issues.

### Flutes and musicology

**Figure 5.**
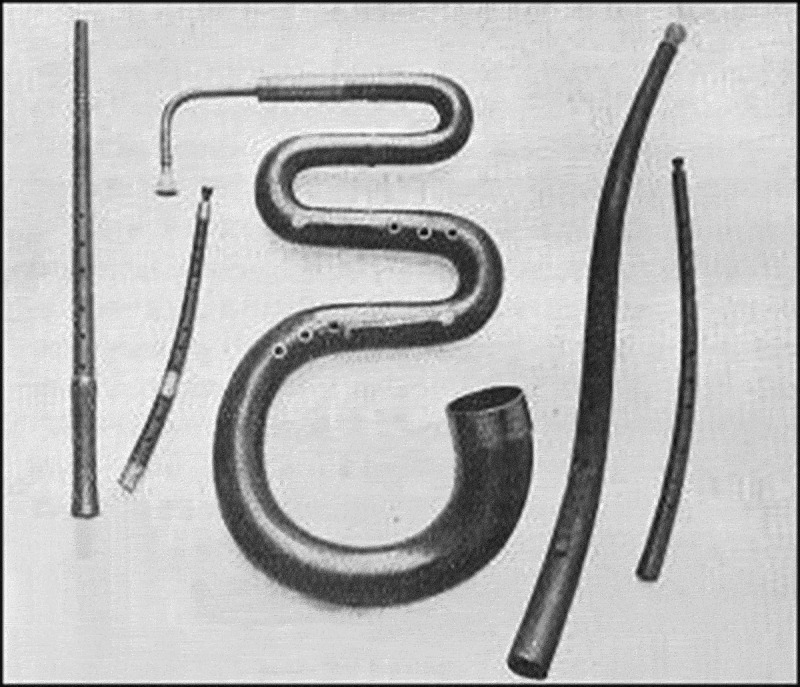
In his essay *Old Instruments of Music* Francis Darwin described and depicted several kinds of flutes etc. This worm-like old instrument is reminiscent to a “lower organism”, as studied by father Charles in his book on the *Earthworms* (adapted from Darwin, F.: *Springtime and Other Essays*, 1920).

It is very likely that Francis Darwin had inherited his drive to become a naturalist from his father Charles, and his interest in music from mother Emma, who was an accomplished pianist. Accordingly, he described his fascination with music in his *Springtime*-Essays as follows (Article “Old Instruments of Music”): ”My own experience of instruments of music is confined to the wind band. I remember as a small boy at school struggling with an elementary flute: or was it a penny whistle? I believe it was a flute, for I have a dim recollection of pouring water into it before it would sound. I tried to teach the instrument – whatever it was – to a friend, and wrote down the fingerings by a series of black and white dots, … Then when I was about fifteen or sixteen years old I began under that admirable teacher, the late R. S. Rockstro, to work regularly at the flute (see Figure 2). As a Cambridge undergraduate I remember playing flute solos at the University Musical Society’s concerts. And I can still recall the pleasant sound of the applause which on one occasion called for a repetition of my performance. Since those days I took up the bassoon under the guidance of another admirable teacher, Mr E. F. James. But nowadays my chief interest is the recorder (i.e., the Blockflöte)” (Figure 5).

Most importantly, Francis Darwin combined his expertise as naturalist/plant biologist and active musician/theoretical musicologist (see, for instance, his Essay on “War Music” in *Rustic Sounds*).^17^ As a result, the “biologist in the shadow of father Charles” compared the emergence and growth of flowers during springtime with the 6^th^ Symphony of Ludwig van Beethoven (1770–1827) in a philosophical way. Accordingly, Francis Darwin was one of the first musicologists capable of analyzing ”Creative” biological processes with respect to the human art of composing-performing classical music.^18^ Finally, son Francis provided a valuable description of the private life and habit of father Charles, when he recollects the two most important “Rustic Sounds” experienced regularly during his years at Down House:

### The sound of father Charles

“One sound there was peculiar to Down,—I mean the sound of drawing water. In that dry chalky country we depended for drinking-water on a deep well from which it came up cold and pure in buckets. These were raised by a wire rope wound on a spindle turned by a heavy fly-wheel, and it was the monotonous song of the turning wheel that became so familiar to us.” … “Another sound I like to recall is connected with the memory of my father. He daily took a certain number of turns round a little wood planted by himself, and christened it the Sandwalk. As he paced round it he struck his heavy iron-shod walking-stick against the ground, and its rhythmical click became a familiar sound that spoke of his presence near us, and was associated with his constant sympathy in our pursuits. It is a sound that seems to me to have lasted all those years that stretch from misty childish days until his death. I am sure that all his children loved that sound.”

The second quote corroborates the well-known fact that Charles Darwin took his daily walk on his private property in Down-Kent, UK – the only exercise documented in the later life of the famous British naturalist.

## Conclusions

Our brief summary of the life and work of the “younger Darwin” raises the following questions: Why remains Sir Francis Darwin (Figures 1, 2), despite his outstanding achievements as plant biologist, musicologist and biographer/philosopher of the natural sciences, 100 years after his death, still in the shadow of father Charles? We think that at least three reasons account for this eclipse of Darwin’s most gifted son.

First, Francis Darwin focused, as a biologist, on plants, whereas father Charles discussed in his books microbes, plants, animals and humans, with a focus on his theoretical 1859-principle of “Descent with modification”—in modern terms, organismic evolution (ref. 29). Hence, Charles Darwin was a generalist/theorist, whereas his son Francis was “only” a specialist in the life sciences. Second, in the *Descent of Man*, Charles Darwin proposed that the species *Homo sapiens* descended from a “lower, ape-like animal”, a conclusion that greatly contributed to the fame of father Charles,^20^ and may have prevented him from receiving the honor of becoming a British “Sir” (knighthood). Finally, Charles Darwin was a gifted “networker” and had influential friends, like Thomas Henry Huxley (1825–1895), who promoted his ideas and conclusions summarized in his *Origin of Species* (1859; 6^th^. Ed. 1872)^19^ among the general public. In addition, it should be noted that the valuable contributions of the “polymath” Sir Francis Darwin were distributed in the form of journal articles, most of them research papers, and public lectures (plus a number of essays). His two books on Plant Biology^16,21^ were written for specialists, not for the general audience. In contrast, father Charles published 16 books on a variety of topics, from his popular “Journal of Researches,1839” to the “Little Earthworm-monograph,1881”. With these publications, plus his great theoretical “Principle of Evolution”, he became one of the most famous generalists of the biological sciences, despite much hostility and jealousy among his numerous enemies. These “Anti-Darwinists” were comprised of Christian fundamentalists/Creationists, naturalistic skeptics of his “Theory of descent with modification via natural selection”, and those who rejected his ideas outlined in the *Power of Movements in Plants*, such as the German biologist Julius Sachs. Finally, we want to stress that the “C. Darwin & F. Darwin” circumnutation-theory of 1880, stating that these endogenous processes may represent the “archetype” of all plant movements, was later rejected by son Francis – a brilliant biologist and philosopher of science (plus musicology), who deserves more recognition than he currently receives.

## Data Availability

The data supporting this study’s findings are available on request from the corresponding author.
